# Resistance training-induced gains in knee extensor strength are related to increased neural cell adhesion molecule expression in older adults with knee osteoarthritis

**DOI:** 10.1186/s13104-019-4642-0

**Published:** 2019-09-18

**Authors:** Thomas B. Voigt, Timothy W. Tourville, Michael J. Falcone, James R. Slauterbeck, Bruce D. Beynnon, Michael J. Toth

**Affiliations:** 1 Department of Medicine, College of Medicine, University of Vermont, Burlington, VT USA; 2 Department of Molecular Physiology and Biophysics, College of Medicine, University of Vermont, Health Science Research Facility 126B, 149 Beaumont Ave, Burlington, VT 05405 USA; 3 Department of Orthopedics and Rehabilitation, College of Medicine, University of Vermont, Burlington, VT USA; 4 Department of Rehabilitation and Movement Science, College of Nursing and Health Sciences, University of Vermont, Burlington, VT USA

**Keywords:** Exercise, Hypertrophy, Denervation, Reinnervation, Aging, Osteoarthritis

## Abstract

**Objective:**

Resistance training (RT) can improve whole muscle strength without increasing muscle fiber size or contractility. Neural adaptations, which lead to greater neural activation of muscle, may mediate some of these improvements, particularly in older adults, where motor neuron denervation is common. The purpose of this study was to explore the relationship of neural adaptations, as reflected by neural cell adhesion molecule (NCAM) expression, to improvements in (1) whole muscle strength and (2) muscle fiber size following RT in older adults with knee osteoarthritis. We performed whole muscle strength measurements and immunohistochemical analysis of fiber size, type, and NCAM expression before and after a 14-week RT program.

**Results:**

RT increased whole-muscle strength as measured by 1-repetition maximum (1-RM) leg press (P = 0.01), leg extension (P = 0.03), and knee extensor peak torque (P = 0.050), but did not alter NCAM expression. Greater NCAM expression in myosin heavy chain (MHC) II fibers was associated with greater whole muscle strength gains (knee extensor peak torque r = 0.93; P < 0.01) and greater MHC II fiber size (r = 0.79; P < 0.01). Our results suggest that training-induced NCAM expression, and neural adaptations more generally, may be important for RT-induced morphological and functional improvements in older adults.

*Trial registration* NCT01190046

## Introduction

Progressive resistance training (RT) increases whole muscle strength in older adults by increasing muscle size and improving myofilament function and neural activation [1]. However, in studies of very old adults (> 80) [2, 3], and older adults with knee osteoarthritis (OA) [4], no improvements in muscle fiber size and myofilament function were found despite improved whole muscle strength. Strength improvements with RT in older adults may be explained, in part, via reinnervation of skeletal muscle fibers, as indicated by decreased neural cell adhesion molecule (NCAM) expression [5]. Such adaptations may be particularly apparent in older adults with advanced stage knee OA, where there are reductions in motor neuron activation secondary to knee pain and arthrosis [6]. That is, because of impaired neural activation in these patients, they may be more likely to experience improvements in muscle neural innervation and/or activation. To explore whether improvements in muscle strength in older adults with knee OA [4] are explained by reinnervation of muscle fibers, we examined the relationship of changes in NCAM expression to changes in whole muscle function. We hypothesized that RT would decrease NCAM expression and that this decrease would be associated with whole muscle strength improvements.

## Main text

### Methods

#### Participants

This report represents additional analysis on a subset of 7 (5 men, 2 women) older adult volunteers from a cohort of patients (n = 17) in a previously published study [4]. Non-obese (BMI < 30 kg/m^2^), older adults (60–80 years) with radiographic and symptomatic evidence for advanced stage knee OA and who reported being inactive or participating in only light activities, were included, as detailed [4]. Volunteers were excluded based on a history, clinical signs or symptoms of diseases that might affect skeletal muscle size, function or functional adaptations to training, as described [4]. Subjects were included in the present study based on availability of muscle tissue for immunohistochemical evaluation [4]. Data on RT-induced adaptations in muscle size and strength at the whole muscle and single fiber level have been reported for the entire cohort studies [4].

#### Experimental protocol

Whole-muscle strength tests were performed on the leg with knee OA and vastus lateralis muscle tissue obtained by percutaneous biopsy, as described [7]. Within 1–3 weeks of biopsy, volunteers started a 14-week moderate-intensity, unilateral, progressive RT program. After a run-in training period of 2 weeks, during which patients visited the training center 3 times per week to learn proper form for each exercise [1 set of 8 repetitions at 30% of 1 repetition maximum (1-RM)] and perform 1-RM testing, volunteers underwent a 12-week regimen with a target intensity of 60% 1-RM. Exercises included leg press, leg curl, leg extension, calf raises, hip extension and hip flexion. Volunteers performed exercises at 40% 1-RM during the first week, 50% 1-RM during the second, and 60% 1-RM for the remaining weeks, with a second set of 8 repetition of each exercise added on the fourth week and maintained throughout the remainder of the program. Reevaluation of 1-RM was performed every 3 weeks throughout the regimen and exercise prescriptions were modified accordingly. Patients were instructed in the proper form of each exercise and monitored throughout the program by the same exercise physiologist. A more moderate intensity program was used because knee pain in these volunteers prohibits higher intensity exercise, which would increase attrition rates. Both legs were trained, but exercise prescriptions were leg specific because of the possibility for strength asymmetries secondary to knee OA, as described [7]. Strength measurements and the muscle biopsy were repeated at the completion of the training program.

#### Knee extensor function

Volunteers underwent isometric (70°) knee extensor torque and one repetition maximum (1-RM) for leg press and leg extension, as described [8, 9]. These two indices were chosen for analysis because they experienced improvements with training [4]. Data are presented on the leg with knee OA only.

#### Muscle biopsy

Baseline and post-training biopsies were performed, as described [10]. Muscle tissue was frozen in embedding medium (OCT; Sakura, Torrence, CA) in liquid N_2_-cooled isopentane, as described [11].

#### Immunohistochemistry

Single fiber morphology and NCAM expression were assessed by immunohistochemistry (IHC), as described [11]. Sections (6 µm) were fixed in ice-cold acetone for 10 min before blocking with 5% goat serum for 1 h and incubating overnight with anti-NCAM antibody (AB5032; Millipore, Temecula, CA), which has been validated in knockout animals [12]. On the second day, sections were blocked with 5% goat serum (1 h) before overnight incubation with anti-myosin heavy chain (MHC) I (BA-D5; DSHB, Iowa City, Iowa) and anti-dystrophin antibodies (MANDRA1[7A10]; DSHB, Iowa City, Iowa). Minimum Feret’s diameter and mean NCAM signal intensity measurements were obtained for each fiber, with the former being an index of cell size that controls for myofiber orientation and cutting artifact [13]. A second slide was prepared for each patient and stained using the same protocol with the exception that the NCAM primary antibody was not included. The exclusion of the NCAM primary in the staining process was used to derive an average background intensity from non-specific binding for each patient, which was subtracted from single fiber NCAM intensity measurements.

#### Statistics

Changes in strength measures were assessed by paired t-tests (SPSS version 25; IBM SPSS Statistics, Armonk, NY). Mixed model analyses were performed for variables with multiple observations within each volunteer (NCAM mean intensity, fiber diameter), using SAS version 9.3 (SAS Institute, Cary, NC), as described [4]. Correlations between variables were derived using parametric statistics (SPSS version 25; IBM SPSS Statistics, Armonk, NY). For variables with multiple observations within each volunteer (NCAM mean intensity, fiber diameter), these were averaged to derive a single value per volunteer for correlation analysis. All data are mean ± SE (n = 7, df = 6), and significance was considered at P < 0.05.

### Results

RT increased whole muscle strength (Table 1) measured using 1-RM leg press (t = − 3.685; P = 0.010) and knee extension (t = − 2.784; P = 0.032), and isometric knee extensor peak torque (t = − 2.45; P = 0.050). Single fiber size did not change when all fibers were pooled, due to reduced diameter in MHC I fibers (t = 4.96; P < 0.01) and increased diameter in MHC II fibers (t = − 5.26; P < 0.01). These results agree with those observed in the entire cohort [4].

**Table 1:** RT-induced changes in strength and immunohistochemical data

Parameter	Pre	Post
n (M/F)	5/2	5/2
Leg press (kg)	53.6 ± 8.6	95.7 ± 16.6*
Leg extension (kg)	30.7 ± 4.4	47.9 ± 8.9*
Knee extensor isometric peak torque (Nm)	129 ± 17.8	141 ± 16.8*
All fibers diameter (μM)	57.4 ± 4.0	55.8 ± 2.2
MHC I diameter (μM)	63.5 ± 2.7	60.1 ± 2.7**
MHC II diameter (μM)	49.5 ± 3.0	53.1 ± 3.0**
NCAM mean intensity (AU)	16.6 ± 4.2	13.9 ± 2.5
NCAM MHC I mean intensity (AU)	10.4 ± 1.9	9.6 ± 1.9
NCAM MHC II mean intensity (AU)	17.7 ± 2.5	17.7 ± 2.5

Values represent mean ± standard error

*Diameter* minimum Feret’s diameter

* P < 0.05

** P < 0.01

**Fig. 1:**
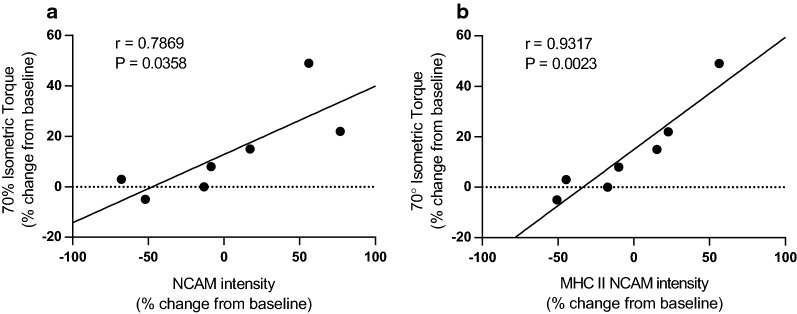
Relationship between NCAM expression and knee extensor 70° isometric peak torque. Overall (**a**) and MHC II (**b**) NCAM intensity show positive linear correlations with knee extensor 70° isometric peak torque following resistance exercise training

**Fig. 2:**
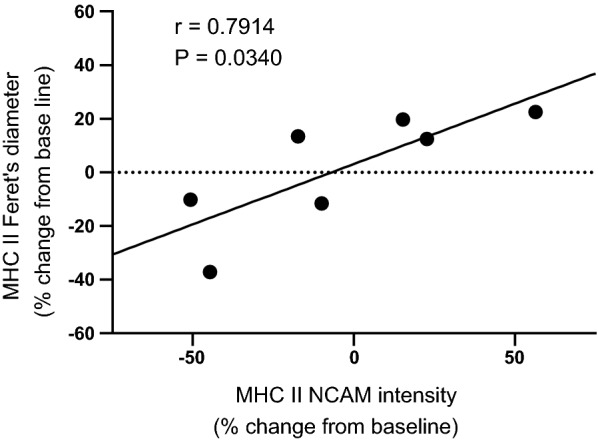
Relationship between minimum Feret’s diameter and NCAM expression. MHC II minimum Feret’s diameter is positively correlated with MHC II NCAM intensity following resistance exercise training

Average NCAM intensity did not change with RT in pooled fibers (P > 0.67) or MHC I (P > 0.13) or MHC II (P > 0.97) fibers separately (Table 1). However, increased average NCAM signal in all fibers pooled together (r = 0.79; P = 0.04), and in MHC II fibers alone (r = 0.93; P < 0.01), were associated with greater isometric knee extensor torque (Fig. 1). Increased NCAM signal in MHC II fibers (r = 0.79; P = 0.03) was also associated with greater MHC II fiber minimum Feret’s diameter (Fig. 2). Increased NCAM signal in MHC I fibers was not correlated with greater MHC I fiber minimum Feret’s diameter (P > 0.10). No correlations were found between NCAM expression and 1-RM data.

### Discussion

Contrary to our hypothesis, there were no changes in NCAM expression with RT. However, there was wide variation in RT-induced changes in NCAM expression, as shown graphically in Figs. 1 and 2. Moreover, we found that greater NCAM expression in all fibers pooled or MHC II fibers alone was related to greater isometric knee extensor torque (Fig. 1). Greater NCAM expression in MHC II fibers was also associated with RT-induced increases in MHC II fiber size. Some have suggested that MHC II fibers are at a greater risk of age-related atrophy [14] and that RT preferentially enhances MHC II fiber function and size [15]. This may explain our finding that NCAM expression in MHC II fibers more prominently correlated with RT-induced improvements in strength and fiber size. Together, these findings suggest that improvements in both fiber diameter and whole muscle strength with RT in older adults with knee OA may be mediated via neural adaptations/muscle fiber reinnervation.

Further conflicting with our original hypothesis, we found positive correlations between NCAM expression and both strength gains and fiber size increases. Our hypothesis was based on recent data showing that a 5-month RT regimen decreased NCAM expression in MHC I fibers in obese older individuals, and that decreased NCAM expression was inversely related to strength gains. The authors interpreted the reduction in NCAM expression as an indicator of fiber reinnervation and/or rescue from denervation [5] and, in turn, that these neural adaptations explain RT-induced strength gains. The disparity in findings between studies could be explained by where each population resides on the muscle disuse continuum. Because of long-standing knee pain related to their OA, our volunteers likely have a more protracted exposure to muscle disuse compared to obese older adults. NCAM expression, or its response to RT, may differ depending on habitual muscle use patterns. Moreover, NCAM expression may not be a perfect index of denervation [16], as denervation can result in increases or decreases in NCAM expression, depending on age and time-point following denervation [16, 17]. In this context, NCAM expression at baseline, or its response to RT, may represent different types of neural adaptations in the two populations. NCAM can undergo post-translational modification via addition of sialic acid monomers to create polysialylated-NCAM [18]. The degree to which NCAM is polysialylated can act as a further regulator of cell–cell contacts [19]. Thus, improved skeletal muscle function following RT may result from alterations in NCAM expression, its PSA content or some combination of these adaptations. Lastly, differences in results may be explained by RT regimen length if NCAM expression varies by training duration. For instance, improved innervation with RT may yield an early increase in NCAM expression to establish new junctional contacts, followed by reductions upon reinnervation. This could explain why we found positive associations between strength gains and NCAM expression in our 14-week training program, when reinnervation may be on-going, and Messi et al. [5] showed negative correlations at 6 months, when reinnervation is more complete.

Regardless of differences between studies, both support a role for neural adaptations in RT-induced improvements in skeletal muscle function and size in older adults and hint at a potentially complex regulation. In this context, therapies that seek to enhance neural adaptations may be an effective adjunct to derive greater benefits from progressive RT programs in older adults. Future research into the relationship between the nervous and musculoskeletal system adaptations may elucidate the mechanisms by which these interventions are successful and aid in the development of new approaches to more effectively combat disability.

## Limitations

- Since NCAM can be associated with both denervation and reinnervation, we cannot definitively know that the improvements we see are due to reinnervation or rescue from denervation. However, that NCAM expression is correlated with strength and fiber size improvements provides sufficient evidence to conclude that neural adaptations are involved in mediating these effects. Whether these improvements are by reinnervation, rescue from denervation, or some other NCAM-mediated neural adaptations remains to be determined in future studies.
- A control group was not included in the study, making it difficult to discern whether the absence of NCAM changes constitute a lack of a training effect. For example, older adults with knee OA may experience declines in strength and fiber size, with concurrent increases in NCAM expression associated with denervation. In this case, no change in NCAM could indicate that RT is countering these changes, preventing and reversing atrophy and denervation.

## Data Availability

The datasets used and/or analyzed during the current study are available from the corresponding author on request.

## Abbreviations

RT: resistance training
OA: osteoarthritis
NCAM: neural cell adhesion molecule
1-RM: 1-repetition maximum
IHC: immunohistochemistry
MHC: myosin heavy chain

## References

[1] Frontera WR, Hughes VA, Krivickas LS, Kim S, Foldvari M, Roubenoff R (2003). Strength training in older women: early and late changes in whole muscle and single cells. Muscle Nerve.

[2] Raue U, Slivka D, Minchev K, Trappe S (2009). Improvements in whole muscle and myocellular function are limited with high-intensity resistance training in octogenarian women. J Appl Physiol.

[3] Slivka D, Raue U, Hollon C, Minchev K, Trappe S (2008). Single muscle fiber adaptations to resistance training in old (> 80 yr) men: evidence for limited skeletal muscle plasticity. Am J Physiol Regul Integr Comp Physiol.

[4] Miller MS, Callahan DM, Tourville TW, Slauterbeck JR, Kaplan A, Fiske BR, Savage PD, Ades PA, Beynnon BD, Toth MJ (2017). Moderate-intensity resistance exercise alters skeletal muscle molecular and cellular structure and function in inactive older adults with knee osteoarthritis. J Appl Physiol.

[5] Messi ML, Li T, Wang Z, Marsh AP, Nicklas B, Delbono O (2016). Resistance training enhances skeletal muscle innervation without modifying the number of satellite cells or their myofiber association in obese older adults. J Gerontol A Biol Sci Med Sci.

[6] Petterson SC, Barrance P, Buchanan T, Binder-Macleod S, Snyder-Mackler L (2008). Mechanisms undlerlying quadriceps weakness in knee osteoarthritis. Med Sci Sports Exerc.

[7] Callahan DM, Miller MS, Sweeny AP, Tourville TW, Slauterbeck JR, Savage PD, Maugan DW, Ades PA, Beynnon BD, Toth MJ (2014). Muscle disuse alters skeletal muscle contractile function at the molecular and cellular levels in older adult humans in a sex-specific manner. J Physiol (Lond).

[8] Ades PA, Savage PD, Brochu M, Tischler MD, Lee NM, Poehlman ET (2005). Resistance training increases total daily energy expenditure in disabled older women with coronary heart disease. J Appl Physiol.

[9] Callahan DM, Bedrin NG, Subramanian M, Berking J, Ades PA, Toth MJ, Miller MS (2014). Age-related structural alterations in human skeletal muscle fibers and mitochondria are sex specific: relationship to single-fiber function. J Appl Physiol.

[10] Toth MJ, Miller MS, VanBuren P, Bedrin NG, LeWinter MM, Ades PA, Palmer BM (2012). Resistance training alters skeletal muscle structure and function in human heart failure: effects at the tissue, cellular and molecular levels. J Physiol (Lond).

[11] Callahan DM, Tourville TW, Miller MS, Hackett SB, Sharma H, Cruickshank NC, Slauterbeck JR, Savage PD, Ades PA, Maughan DW, Beynnon BD, Toth MJ (2015). Chronic disuse and skeletal muscle structure in older adults: sex-specific differences and relationships to contractile function. Am J Physiol Cell Physiol.

[12] Watzlawik JO, Kahoud RJ, Ng S, Painter MM, Papke LM, Zoecklein L, Wootla B, Warrington AE, Carey WA, Rodriguez M (2015). Polysialic acid as an antigen for monoclonal antibody HIgM12 to treat multiple sclerosis and other neurodegenerative disorders. J Neurochem.

[13] Briguet A, Courdier-Fruh I, Foster M, Meier T, Magyar JP (2004). Histological parameters for the quantitative assessment of muscular dystrophy in the mdx-mouse. Neuromuscul Disord.

[14] Lexell J, Henriksson-Larsén K, Winblad B, Sjöström M (1983). Distribution of different fiber types in human skeletal muscles: effects of aging studied in whole muscle cross sections. Muscle Nerve.

[15] Charette SL, McEvoy L, Pyka G, Snow-Harter C, Guido D, Wiswell RA, Marcus R (1991). Muscle hypertrophy response to resistance training in older women. J Appl Physiol.

[16] Grumbles RM, Almeida VW, Thomas CK (2008). Embryonic neurons transplanted into the tibial nerve reinnervate muscle and reduce atrophy but NCAM expression persists. Neurol Res.

[17] Hendrickse P, Galinska M, Hodson-Tole E, Degens H (2018). An evaluation of common markers of muscle denervation in denervated young-adult and old rat gastrocnemius muscle. Exp Gerontol.

[18] Figarella-Branger D, Nedelec J, Pellissier JF, Boucraut J, Bianco N, Rougon G (1990). Expression of various isoforms of neural cell adhesive molecules and their highly polysialylated counterparts in diseased human muscles. J Neurol Sci.

[19] Rutishauser U, Watanabe M, Silver J, Troy FA, Vimr ER (1985). Specific alteration of NCAM-mediated cell adhesion by an endoneuraminidase. J Cell Biol.

